# Intranasal biomaterials for treatment-resistant depression: device–formulation coupling and therapeutic exposure

**DOI:** 10.3389/fbioe.2026.1913962

**Published:** 2026-08-13

**Authors:** Wentao Wu, Jiali He, Jiaxin Hu, Xin Xu, Mingzhe Yang, Fei Wen

**Affiliations:** The Second Affiliated Hospital of Guangzhou Medical University, Guangzhou, China

**Keywords:** bioengineering, brain pharmacokinetics, digital outcome assessment, intranasal drug delivery, machine learning, nasal spray device, nose-to-brain delivery, regional deposition

## Abstract

Treatment-resistant depression (TRD) is commonly defined as depression that does not achieve an adequate response or remission after at least two antidepressant treatments of adequate dose and duration during the current depressive episode, although exact criteria vary across studies. Intranasal delivery is attracting renewed interest in TRD because it enables rapid administration, avoids gastrointestinal degradation and first-pass metabolism, and may support direct or indirect access to the central nervous system. However, clinical translation cannot be established by particle size, drug loading, or behavioral efficacy alone. A viable intranasal biomaterial–device product must generate reproducible therapeutic exposure through a coupled formulation–device–anatomy system, in which material properties shape spray performance, regional deposition, mucosal residence, epithelial transport, pharmacokinetics, safety, and therapeutic response. This Perspective examines how polymer composition, surface chemistry, particle mechanics, gelation, viscosity, and release kinetics interact with metered-dose devices, spray plume characteristics, nasal anatomy, and mucociliary clearance. Particular attention is given to the distinction between nasal deposition and brain delivery, the need for quantitative pharmacokinetic and biodistribution evidence, and the local safety requirements of repeated administration to respiratory and olfactory epithelia. We also propose indication-specific outcome standards for treatment-resistant depression that extend beyond short-term behavioral tests or isolated symptom scores to include onset, durability, cognition, sleep, daily function, adherence, and treatment burden. Artificial intelligence, medical-image-based airflow simulation, three-dimensional nasal models, and digital outcome measures may strengthen selected steps of this development pathway, but they should support rather than replace experimental validation. The central position of this article is that intranasal biomaterial–device products for treatment-resistant depression should be judged by reproducible, clinically relevant exposure with an acceptable benefit–risk profile, rather than by material sophistication alone.

## 1 Introduction

Treatment-resistant depression (TRD) is most commonly operationalized as failure to achieve adequate response or remission after at least two antidepressant trials of adequate dose and duration within the current depressive episode, although diagnostic thresholds, minimum treatment duration, augmentation strategies, and definitions of response vary across studies (Gaynes et al., 2020). It therefore remains a clinically heterogeneous condition rather than a single pharmacological entity. Patients can reach the same treatment-resistant designation through different combinations of inadequate efficacy, delayed response, dose-limiting adverse effects, poor adherence, comorbidity, and incomplete functional recovery (Mickey et al., 2018). The declining probability of remission across successive treatment steps is well established, but the usual explanation - that the next drug must engage a better molecular target - captures only part of the problem (McIntyre et al., 2021). A therapy may also fail because the active compound reaches the relevant brain compartment too slowly, too briefly, or too variably to produce a reliable effect. In this manuscript, therapeutic exposure is operationally defined as the integrated profile of dose amount, timing, regional distribution, chemical integrity, and inter- or intra-subject variability of the active compound or functional carrier in nasal tissues, systemic circulation, and relevant brain compartments, interpreted together with target engagement, safety, and therapeutic response.

The clinical use of intranasal esketamine shows that the nasal route can support rapid antidepressant treatment, while also illustrating that an intranasal antidepressant product is inseparable from its delivery device, administration instructions, observation requirements, and safety controls (Daly et al., 2018). The approved product is a metered drug-device combination rather than a free-standing molecule, and its dosing schedule is adjusted according to efficacy and tolerability. This precedent has stimulated interest in nanoparticles, lipid carriers, nanoemulsions, nanogels, and in situ-forming hydrogels for other antidepressant cargos. Preclinical studies have reported enhanced brain delivery or antidepressant-like activity for intranasal formulations of venlafaxine, paroxetine, berberine, albiflorin, and experimental anti-inflammatory combinations (Carboni et al., 2019; Xu et al., 2020; Kargbo, 2025). To avoid ambiguity, we use “intranasal biomaterial–device product” as the primary term for the integrated therapeutic system comprising the active cargo, biomaterial carrier or matrix, excipients, and delivery device. When referring specifically to the carrier or matrix independent of the device, we use “intranasal biomaterial formulation.” The term “intranasal nanomedicine” is retained only when discussing nanoscale systems reported in the literature. Because TRD-specific evidence for biomaterial–device intranasal products remains limited, we use selected examples from general depression, other central nervous system disorders, and broader intranasal delivery research to illustrate engineering and delivery-related principles, including deposition, mucosal residence, epithelial transport, pharmacokinetics, biodistribution, and safety. These examples should therefore be interpreted as mechanistic or translational design evidence rather than as direct clinical evidence for TRD.

Despite this activity, the evidence base remains fragmented. Formulation studies frequently optimize particle size or entrapment efficiency without characterizing how the final product behaves during spraying. Deposition studies may report total nasal retention without distinguishing anterior loss from delivery to upper nasal regions. Brain-distribution studies sometimes infer direct nose-to-brain transport from a higher brain concentration after intranasal dosing without adequately separating swallowed, vascular, lymphatic, and perineural contributions (Patel and Wairkar, 2025). Behavioral improvement may be reported without exposure-response analysis, target engagement, or repeated-dose nasal safety. These gaps are not minor technical omissions; they prevent a clear account of what dose was delivered, where it travelled, and why it worked.

**FIGURE 1:**
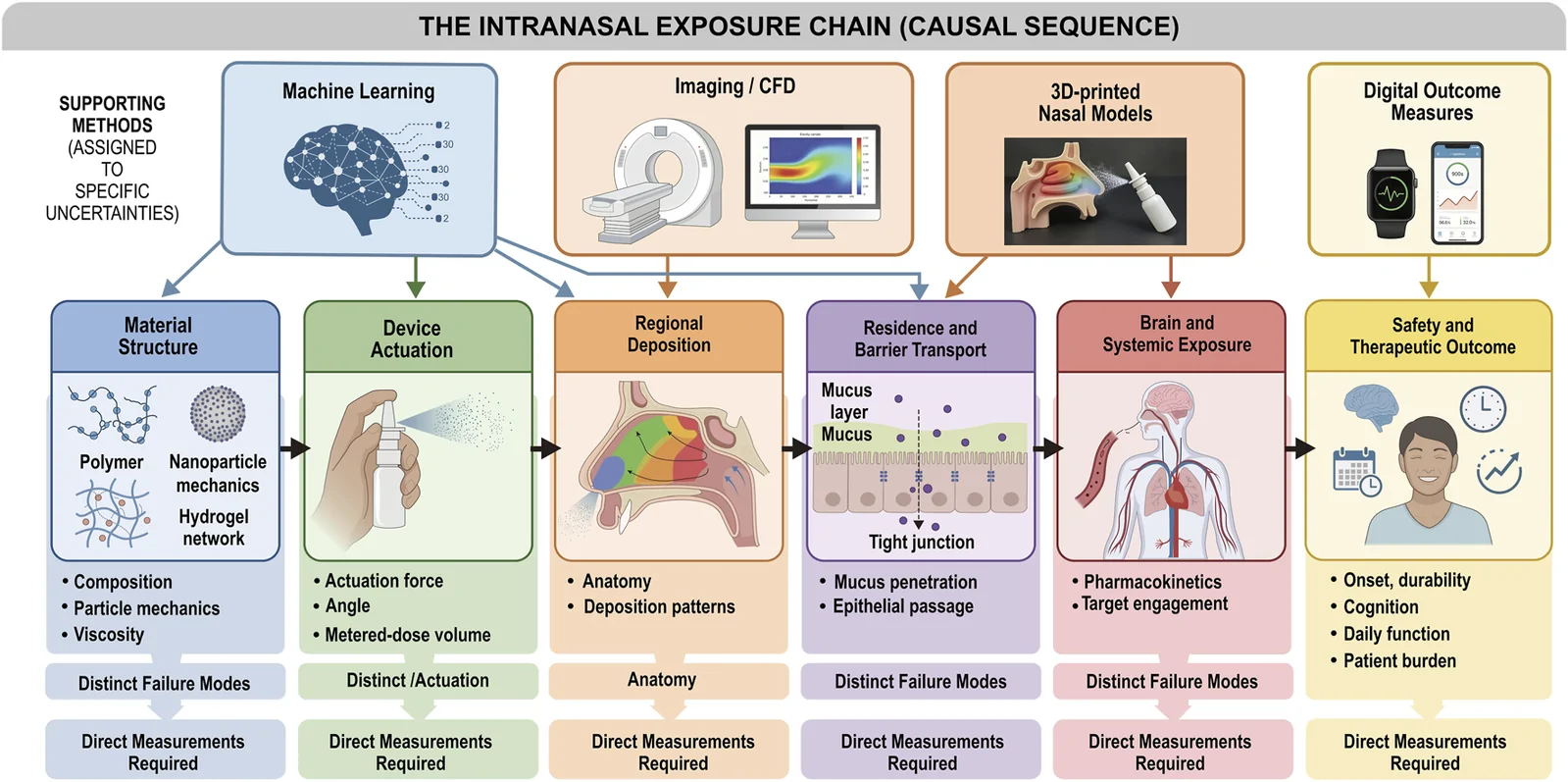
The intranasal exposure chain for treatment-resistant depression. Material structure, device actuation, regional deposition, residence and barrier transport, brain and systemic exposure, and safety and therapeutic outcome form an interdependent translational sequence. Each step has a distinct failure mode and requires direct measurement. Machine learning, imaging/CFD, 3D-printed nasal models, and digital outcome measures are supporting methods assigned to specific uncertainties rather than components of a general closed-loop architecture.

This Perspective therefore uses the intranasal exposure chain, as illustrated in Figure 1, as its organizing principle. The relevant unit of development is the formulation-device-exposure system: material structure influences spray and transport behavior; the device and user action contribute to the administered dose; anatomy shapes regional deposition; mucus and epithelium affect residence and passage; pharmacokinetics inform target exposure; and safety and therapeutic outcomes support judgments of clinical value. The original conceptual contribution of this article is not to provide another catalogue of intranasal carrier types, excipients, or nose-to-brain transport routes. Instead, it proposes an exposure-engineering framework that connects biomaterial design, device–formulation coupling, regional deposition, pharmacokinetics, safety, and indication-specific outcomes for treatment-resistant depression. Artificial intelligence (AI), computational fluid dynamics (CFD), medical imaging, three-dimensional (3D) nasal models, and digital measures are considered as tools that may reduce uncertainty at specific links in this framework, rather than as a general closed-loop architecture. This narrower engineering focus strengthens the scientific and translational value of the framework by linking material design decisions to measurable exposure, safety, and therapeutic outcomes.

## 2 The administered nasal dose is a formulation-device-anatomy product

### 2.1 Formulation mechanics and device actuation

A nominal dose printed on a formulation label is not necessarily the dose that reaches the nasal mucosa. The administered dose is created when a formulation passes through a particular device under a particular actuation force and angle. Viscosity, surface tension, density, thixotropy, elasticity, particle concentration, and gelation kinetics can alter pump filling, plume geometry, droplet-size distribution, spray pattern, and the fraction retained within the actuator (Inthavong et al., 2025). These effects are especially relevant for nanoparticle suspensions and thermoresponsive systems, which may behave differently from low-viscosity solutions.

This device-formulation coupling produces several clinically important trade-offs. Increasing viscosity may reduce posterior drainage and prolong mucosal contact, but it can also produce a narrower plume, larger droplets, incomplete atomization, and dose non-uniformity. Mucoadhesive polymers may improve retention while increasing stringiness or nozzle fouling. A formulation that gels too close to room temperature may undergo premature viscosity change during storage or actuation; one that gels too slowly may drain before a network forms (Wang et al., 2021). Nanoparticles may aggregate under shear, ionic exposure, or repeated temperature cycling, changing both the emitted dose and biological performance (Sipos et al., 2025).

For this reason, spray characterization should be conducted on the final combination product, not on the formulation and device separately. Recommended assessments include delivered-dose uniformity, priming and re-priming behavior, plume geometry, spray pattern, droplet or particle-size distribution, tail-off, residual dose, and performance after storage and transport. These tests should be repeated across clinically realistic actuation forces and device orientations. A material that performs well only with laboratory pipetting has not yet demonstrated intranasal deliverability.

### 2.2 Nasal anatomy makes regional deposition variable

The human nasal cavity is a narrow, asymmetric, highly folded airway. The vestibule, nasal valve, turbinates, respiratory mucosa, olfactory region, and nasopharynx differ in geometry, airflow, epithelial phenotype, mucus dynamics, vascularization, and neural connections (Brüning et al., 2020). Consequently, total nasal retention is a poor surrogate for therapeutically relevant deposition. A large fraction of a conventional spray may remain in the anterior cavity, impact the septum or inferior turbinate, or pass posteriorly and be swallowed. These outcomes may be acceptable for local rhinitis treatment but are not equivalent to reproducible delivery toward upper nasal or olfactory regions (Rigaut et al., 2025).

Device angle, insertion depth, head position, inspiratory flow, nostril patency, septal deviation, turbinate hypertrophy, prior surgery, and mucosal congestion can all shift regional deposition. Numerical and experimental studies show that changing spray position and orientation can materially alter the fraction reaching intended regions (Basu et al., 2020). Patient-derived models extend this analysis by reconstructing nasal geometry from computed tomography (CT) or magnetic resonance imaging (MRI) and testing deposition under controlled conditions. A recent sectioned 3D-printed human nasal cavity model demonstrated how regional recovery can discriminate between spray devices and administration angles (Seifelnasr et al., 2025).

These tools are most useful when treated as product-development instruments rather than as claims of whole-patient digital twins. Imaging-based CFD can identify candidate nozzle positions and flow conditions; 3D-printed models can then test whether those predictions survive real spray behavior, wall wetting, and formulation-specific properties (Jiang et al., 2022; Li J. et al., 2025). The practical objective is not to reproduce every anatomical detail, but to determine whether a formulation-device combination has a robust deposition window across representative nasal geometries.

## 3 Material properties influence residence, mucus transport, and epithelial passage

### 3.1 Mucoadhesion and mucus penetration solve different problems

After deposition, the formulation enters a rapidly renewed mucus layer and is exposed to mucociliary clearance. Extending residence time is therefore a central objective, but retention and transport are not interchangeable. Mucoadhesive polymers such as chitosan and alginate can increase contact with the epithelium through electrostatic, hydrogen-bonding, and chain-interpenetration mechanisms (Menshutina et al., 2022). Their benefit is greatest when rapid drainage is the dominant loss pathway. Excessive adhesion, however, can immobilize a carrier in superficial mucus, limit diffusion toward the epithelial surface, interfere with ciliary function, or increase local irritation (Li S. et al., 2026).

Mucus-penetrating strategies pursue the opposite objective: reducing adhesive interactions to facilitate movement through the mucin network. Small size, near-neutral surface charge, hydrophilic coatings, and controlled protein adsorption may improve diffusion, but weak interaction can also shorten residence and increase clearance (Maeng and Lee, 2022). The correct balance depends on the intended transport route, cargo stability, deposition region, and release time. A rapidly acting small molecule may benefit from prompt epithelial access, whereas a fragile peptide or sustained anti-inflammatory strategy may require a protected depot.

The field would benefit from abandoning generic claims that a material is ‘mucoadhesive’ or ‘mucus-penetrating’ and instead reporting quantitative performance under biorelevant conditions. Useful assays include rheology with nasal mucus or mucin, multiple-particle tracking, wash-off under controlled flow, residence in excised or engineered nasal tissue, ciliary beat frequency, transepithelial electrical resistance, permeability, and recovery of intact cargo (Fieux et al., 2021; Wei et al., 2025). These measurements should ideally be linked to formulation concentration and dose rather than presented only as normalized laboratory indices.

### 3.2 Polymeric, lipid, and hybrid carriers create distinct exposure profiles

Polymeric nanoparticles provide strong control over cargo protection, degradation, and release. Poly(lactic-co-glycolic acid) (PLGA) systems have been explored for venlafaxine and paroxetine, including surface modification intended to improve nasal interaction or transport (Cayero-Otero et al., 2025). Their performance depends on polymer molecular weight, lactide:glycolide ratio, end-group chemistry, particle size, porosity, drug distribution, and residual solvent. These variables influence burst release, erosion, storage stability, and the possibility that an initially uniform formulation changes after contact with mucus.

Lipid nanoparticles, nanoemulsions, liposomes, and transferosomes are attractive for hydrophobic cargos and membrane interaction (Costa et al., 2021). Their fluidity and composition can improve solubilization and epithelial passage, but oxidation, hydrolysis, cargo leakage, surfactant irritation, and temperature-dependent phase behavior complicate translation. A quality-by-design study of intranasal lipid nanoparticles for depression illustrates how formulation and process variables can be linked to particle attributes and permeability rather than optimized one at a time (Albash et al., 2025). Transferosomal gel formulations can combine deformable vesicles with prolonged residence, although the gel may alter vesicle release and effective deformability.

Hybrid systems, particularly nanoparticles dispersed within thermoresponsive or mucoadhesive hydrogels, are often presented as a way to combine penetration with retention. This logic is plausible but creates a two-stage delivery problem: the network must first deposit and remain at the desired site, then release particles or dissolved cargo at a rate that preserves their intended transport function. Alginate-nanogel thermosensitive systems and berberine *in situ* gels have shown improved antidepressant-like effects in animal models (Xu et al., 2025). Translational interpretation requires component controls, however, because the hydrogel, nanoparticle, cargo, and excipients may each affect exposure and biological response.

### 3.3 Responsive behavior should be supported by functional evidence

Terms such as smart, responsive, or intelligent are increasingly applied to intranasal materials. Temperature-triggered gelation is the most directly relevant example because a liquid can be actuated through a device and form a higher-viscosity depot at nasal temperature (van Doorn et al., 2017). Other proposed triggers include pH, ions, enzymes, reactive oxygen species, and inflammatory mediators. These concepts are scientifically relevant to advanced intranasal biomaterials, but the label ‘responsive’ should be reserved for systems in which the stimulus-response-function relationship is measured.

Recommended characterization includes the physiological stimulus range, response threshold, kinetics, reversibility, and effect on transport or release. The decisive control is not a visually convincing sol-gel transition; it is evidence that the response changes regional residence, cargo availability, epithelial passage, or therapeutic exposure under relevant conditions. If a material responds only at stimulus concentrations not present in the nasal cavity, or if the response occurs after the formulation has already cleared, such responsiveness is unlikely to provide clinically meaningful functionality.

## 4 Regional deposition is only the beginning of brain exposure

### 4.1 Multiple transport routes operate after nasal administration

Nasal delivery can contribute to brain exposure through several overlapping routes. Drug may cross respiratory or olfactory epithelium, move extracellularly along olfactory or trigeminal pathways, undergo neuronal uptake and intracellular transport, enter local blood or lymphatic circulation, or be swallowed and absorbed gastrointestinally (Kashyap and Shukla, 2019). These processes differ greatly in speed and efficiency, and their contribution depends on molecular size, lipophilicity, charge, carrier stability, enzymatic susceptibility, and deposition region (Natsheh and Touitou, 2018).

A higher brain concentration after intranasal administration does not, by itself, establish direct nose-to-brain transport. Direct transport may coexist with rapid systemic absorption, and a carrier can disassemble before its cargo reaches neural pathways. Conversely, low whole-brain concentration may obscure high exposure in a therapeutically relevant region. The analytical question is therefore not simply ‘did the drug reach the brain?’ but ‘in what chemical form, through which route, at what time, and in which region did therapeutically active exposure occur?'

### 4.2 Pharmacokinetics, biodistribution, and target engagement should be linked where feasible

A credible exposure package begins with simultaneous plasma, nasal tissue, olfactory bulb, brain-region, and, where feasible, cerebrospinal-fluid measurements across an early and extended time course. Both parent drug and relevant metabolites should ideally be quantified. Brain-targeting indices can be informative, but they should not replace absolute concentrations and uncertainty estimates. Fluorescent carrier tracking is useful only when label stability and separation from released dye are established. Radiolabeling, mass spectrometry imaging, microdialysis, or validated quantitative imaging may be needed when the spatial question is central.

The next step is pharmacodynamic linkage. Antidepressant-like behavior without target engagement cannot distinguish a central mechanism from systemic stress, sedation, locomotor effects, or nonspecific inflammation. Depending on the cargo, target engagement may include receptor occupancy, downstream signaling, synaptic-plasticity markers, neuroimmune changes, electrophysiology, or circuit-level readouts. Recent venlafaxine-PLGA work is important because it focuses on onset after nose-to-brain administration rather than merely reporting a formulation characteristic (Cayero-Otero et al., 2025). Biomineralized silk-fibroin nanoparticles have similarly been evaluated in relation to neuroinflammation and depressive-like behavior, illustrating the value of connecting material performance with a specified biological mechanism (Zhao et al., 2015; Bossi et al., 2021).

Exposure-response analysis should be planned prospectively. If two formulations produce similar behavioral effects despite markedly different brain concentrations, the assumed exposure mechanism requires reconsideration. If higher exposure produces no additional benefit but increases nasal or systemic toxicity, the design objective should shift from maximizing brain uptake to identifying a therapeutic window.

### 4.3 Reproducibility matters more than a single high-exposure result

Intranasal nanomedicine studies often report mean exposure under tightly controlled administration by trained researchers. Clinical use is more variable. Differences in actuation, head angle, congestion, mucus volume, and inhalation can generate large within- and between-patient variation (Patel et al., 2018). Reproducibility should therefore be treated as a primary product attribute rather than a statistical afterthought.

Preclinical studies can begin to address this by testing multiple administration operators, anatomies, and device conditions (Mahajan et al., 2025). Clinical pharmacology studies should characterize within-subject and between-subject variability, identify covariates, and determine whether the device or instructions can narrow the distribution. A formulation that produces a high mean brain-targeting index with wide variance may be less useful than a modest but predictable exposure.

## 5 Repeated-dose safety defines clinical feasibility

### 5.1 Local nasal toxicity cannot be reduced to cell viability

TRD treatment may require induction and maintenance dosing over months or longer. Repeated exposure therefore makes local nasal safety central. Standard cytotoxicity assays are insufficient because the relevant tissue functions include ciliary motion, mucus production, tight-junction integrity, epithelial renewal, olfactory neuron support, vascular reactivity, and local immune defense. Cationic polymers, penetration enhancers, surfactants, preservatives, degradation products, and hyperosmolar formulations can alter these functions even when short-term cell viability remains acceptable (Tada et al., 2018).

Recommended local-safety assessments include histology of respiratory and olfactory regions, ciliary beat frequency, mucociliary transport, epithelial barrier measures, inflammatory markers, mucus and goblet-cell changes, and recovery after dosing stops (Jiao and Zhang, 2018). Olfactory function deserves separate assessment because olfactory epithelium is both a potential access site and a vulnerable neural tissue. The study duration should reflect the proposed clinical schedule, and dose margins should be defined for the complete formulation, including excipients (D’Souza et al., 2026).

### 5.2 Systemic and central safety remain relevant

Nose-to-brain delivery does not eliminate systemic exposure. Highly vascular nasal mucosa can produce rapid plasma concentrations, and swallowed drug can contribute additional absorption (Kumar et al., 2016). For centrally active compounds, rapid peaks may change sedation, dissociation, cardiovascular effects, abuse potential, or cognitive risk (Lokhande and Chinnaiah, 2025). The 2025 prescribing information for esketamine illustrates that a nasal antidepressant may require structured monitoring and a risk-management pathway because route convenience does not remove pharmacological risk (Veraart et al., 2025).

Nanocarrier safety adds further questions: persistence, degradation, complement activation, immune recognition, unexpected tissue distribution, and interaction with concomitant drugs (Piscatelli et al., 2021). Surface modification intended to improve epithelial transport can alter protein adsorption and systemic biodistribution. Long-term development should therefore include toxicokinetics of both cargo and carrier components, not simply behavioral observation or routine serum chemistry.

### 5.3 Manufacturing and device compatibility are safety variables

Batch-to-batch variation in size distribution, free-drug fraction, residual solvent, endotoxin, sterility, viscosity, and device output can change both exposure and toxicity. These variables become more difficult to control in hybrid nanoparticle-hydrogel systems (Gao et al., 2016). Container closure, pump materials, extractables and leachables, microbial preservation, and dose-counter performance should ideally be assessed early rather than only after efficacy has been optimized.

A practical rule is that every material attribute used to explain efficacy should have a corresponding release or stability test. If gelation temperature is essential, it should remain within specification throughout shelf life. If surface charge is essential, it should be measured after storage and after device passage. If a ligand is claimed to support transport, ligand density and functional activity should be controlled at scale.

## 6 Therapeutic evaluation needs indication-specific endpoints

### 6.1 Preclinical studies require stronger behavioral and mechanistic design

Rodent behavioral tests are useful for early screening but can overstate therapeutic specificity (Mohamed-Fathy Kamal et al., 2025). Open-field, tail-suspension, forced-swim, and sucrose-preference results are sensitive to locomotion, stress, handling, sex, strain, circadian timing, and sedative or stimulant effects (Ueno et al., 2022). A convincing preclinical package should use more than one disease-relevant model, prespecified behavioral domains, blinded analysis, exposure confirmation, and controls that separate intranasal administration, carrier effects, and active cargo.

The indication also matters. TRD is not reproduced by a single stress model. Studies should justify whether they are modeling delayed response, non-response after prior antidepressant exposure, inflammation-associated symptoms, anhedonia, cognitive dysfunction, or rapid rescue (Pereira et al., 2016). The formulation should be compared with an exposure-matched conventional route where possible. Otherwise, improved behavior may reflect higher total dose rather than a unique advantage of the intranasal material.

### 6.2 Clinical benefit extends beyond rapid symptom reduction

Clinical development should distinguish onset, magnitude, durability, and functional relevance. A rapidly acting intranasal product may be valuable if it reduces symptoms within hours or days, but repeated supervised administration, transient adverse effects, or short durability may offset that advantage. Core outcomes should include validated depression scales, remission and response, time to benefit, relapse, cognitive and psychomotor function, sleep, quality of life, daily activity, work or social participation, treatment adherence, and patient-reported burden.

The comparison should be clinically meaningful. A novel carrier must demonstrate an advantage over the same cargo in a simpler nasal formulation, the same drug by another route, or an accepted TRD strategy (Phillips et al., 2020). Material complexity is justified only if it improves a decision-relevant endpoint such as onset, dose reduction, tolerability, durability, convenience, or access.

### 6.3 Digital measures are supportive endpoints, not the control architecture

Wearables and smartphones can provide longitudinal information about sleep timing, activity, psychomotor behavior, speech, cognition, and variability between clinic visits (Divya and Kumar, 2025). Such measures may detect early functional change or treatment burden that is missed by episodic scales (Yang et al., 2025). Their role in this development pathway is supportive: to validate whether a reproducible material exposure produces meaningful change in daily life.

Digital endpoints require verification, analytical validation, and clinical validation for the intended context of use (Turner et al., 2025). Device changes, missing data, low adherence, and population bias must be reported. An exploratory activity signal should not be interpreted as efficacy unless it is linked to symptoms, function, and clinical decision-making. This limited role avoids turning an intranasal formulation article into a broad digital-psychiatry framework.

## 7 Computational tools can shorten, but not replace, experimental development

### 7.1 Machine learning is most useful within a controlled formulation space

The number of possible combinations of polymers, lipids, excipients, process conditions, particle attributes, and device parameters quickly exceeds what can be explored by one-factor-at-a-time experiments. Machine learning and Bayesian optimization can prioritize experiments, model nonlinear interactions, and identify formulations that balance competing objectives (Maharjan et al., 2024; Hossain and Sağ, 2026). Recent work in lipid nanoparticle development demonstrates the feasibility of learning quality attributes from composition and process variables and then testing predictions prospectively (Li H. et al., 2025).

For intranasal depression products, useful model outputs include delivered-dose uniformity, sprayability, physical stability, mucus diffusion, epithelial permeability, release kinetics, and toxicity (Suzuki et al., 2020). Brain concentration alone is a poor optimization target because it can reward unsafe or irreproducible designs. Models should be trained on standardized raw data that include failed formulations, assay uncertainty, and manufacturing conditions. Prospective confirmation with newly prepared batches is essential.

### 7.2 Imaging, CFD, and 3D models address the deposition question

Medical imaging and CFD are well suited to problems that are difficult to solve by intuition, such as how nozzle angle, inhalation, particle inertia, and nasal geometry affect regional deposition. Their predictions are strongest when paired with realistic spray characterization and experimental models. 3D-printed nasal replicas can provide region-specific mass recovery and allow systematic comparison of devices, angles, and formulations (Jodat et al., 2020).

These methods should remain bounded to the physical questions they can answer. A CFD model does not establish epithelial permeability or brain uptake. A 3D model cannot reproduce mucus renewal, vascular absorption, neural transport, or inflammation unless additional biological modules are validated. Their proper role is to reduce uncertainty in the administered and deposited dose before animal or clinical studies.

### 7.3 Computational claims need clear guardrails

Any computational contribution should state the decision it supports, its input domain, its uncertainty, and the experiment used to test it. Models trained on one device or one formulation family should not be assumed to generalize to another. Interpretability is valuable when it reveals actionable design relationships, such as the viscosity range at which residence improves before atomization fails (Wang et al., 2022). It is less useful when a complex model merely ranks formulations without a reproducible experimental explanation.

This Perspective therefore does not propose a whole-patient digital twin, autonomous dose adjustment, or a closed-loop psychiatric platform. Such concepts may be valuable elsewhere, but they are not required to address the central bioengineering problem considered here: creating a reproducible intranasal exposure and testing whether it supports a safe therapeutic effect.

## 8 A staged evidence sequence for intranasal biomaterials in TRD

A development program can be organized as a series of gates rather than as parallel demonstrations of material novelty and behavioral efficacy, following the framework detailed in Table 1. Gate 1 establishes formulation identity, stability, and manufacturability. Gate 2 establishes device compatibility and emitted-dose performance. Gate 3 establishes regional deposition and residence. Gate 4 measures mucus transport, epithelial interaction, and cargo integrity. Gate 5 quantifies systemic and brain pharmacokinetics, biodistribution, and target engagement. Gate 6 evaluates repeated-dose local and systemic safety. Gate 7 tests indication-specific efficacy and functional outcomes. Failure at an early gate should redirect material design before expensive downstream studies proceed.

**TABLE 1:** Evidence matrix for an intranasal biomaterial-device product.

Exposure-chain link	Critical variables	Minimum evidence	Failure signal
Material identity and stability	Composition, size/polydispersity index (PDI), charge, loading, free-drug fraction, viscosity, gelation, degradation, storage	Orthogonal characterization; batch reproducibility; stability-indicating assays; cargo integrity	Property drift, aggregation, burst release, uncontrolled degradation, non-scalable process
Device actuation	Metered volume, pump compatibility, force, angle, plume, spray pattern, droplet distribution, residual dose	Delivered-dose uniformity; plume/spray analysis; repeated actuation; storage and transport testing	Incomplete atomization, nozzle fouling, dose variability, dependence on unrealistic actuation
Regional deposition and residence	Anatomy, head position, insertion depth, airflow, viscosity, mucoadhesion, clearance	Imaging/CFD plus experimental nasal model; region-specific recovery; residence and wash-off	Anterior impaction, posterior loss/swallowing, rapid clearance, high anatomical sensitivity
Mucus and epithelial transport	Mucin interaction, diffusion, ciliary function, permeability, carrier integrity, enzymatic stability	Multiple-particle tracking; ciliary beat; barrier integrity; *ex vivo*/engineered tissue transport	Mucus trapping, epithelial injury, cargo degradation, transport only at non-physiological doses
Brain and systemic exposure	Route contribution, plasma PK, regional brain concentration, metabolites, target engagement	Time-resolved plasma/nasal/brain pharmacokinetics (PK); validated tracer; biodistribution; pharmacodynamics	Brain claim based only on fluorescence or one time point; no target engagement; high variability
Safety and therapeutic outcome	Repeated dosing, olfactory/respiratory toxicity, central nervous system (CNS)/systemic effects, onset, durability, function	Repeated-dose histology and function; toxicokinetics; indication-specific efficacy; functional/digital outcomes	Ciliary or olfactory injury, narrow safety margin, efficacy without exposure linkage, excessive treatment burden

The sequence also clarifies what can be claimed at each stage. Particle characterization supports a formulation claim, not a brain-targeting claim. Upper nasal deposition supports anatomical targeting, not direct neural transport. A favorable brain-to-plasma ratio supports exposure selectivity, not therapeutic benefit. Behavioral improvement supports biological activity, not clinical feasibility. A credible translational case emerges only when these claims are connected by measured evidence.

This gate-based approach clarifies the scientific and translational roles of advanced materials, machine learning, medical imaging, three-dimensional manufacturing, and digital health within intranasal biomaterial development. Advanced materials remain the therapeutic core; machine learning may improve formulation and process selection; medical imaging and three-dimensional manufacturing can support deposition testing; and digital health can enrich outcome validation (Squelch, 2018; Mathews et al., 2019; Bannigan et al., 2021). The key value of this framework is that each technology is assigned a defined technical task within the exposure pathway, thereby improving the interpretability, reproducibility, and translational readiness of intranasal biomaterial–device products.

## 9 Discussion

Rather than serving as a conventional overview of intranasal drug-delivery systems, this Perspective reframes intranasal biomaterial development for treatment-resistant depression as an exposure-engineering problem. Intranasal biomaterial–device products for depression are often presented as a route to bypass the blood-brain barrier, increase brain uptake, and accelerate antidepressant action. These goals are compelling, but they can obscure a more basic question: what is the actual therapeutic dose created by the formulation, device, anatomy, mucus, epithelium, and transport pathways? Without a quantitative answer, an apparently effective nanocarrier may be difficult to reproduce, scale, or compare with simpler alternatives.

The exposure-chain perspective changes how success is judged. The first requirement is not maximal loading or the smallest particle. It is a stable product that can be emitted reproducibly. The second is not total nasal retention, but deposition in a region consistent with the proposed route. The third is not fluorescence in the brain, but quantitative exposure to intact active compound with a plausible route and time course. The fourth is not a single behavioral improvement, but a linked pharmacokinetic, mechanistic, safety, and therapeutic response. The fifth is not technological integration for its own sake, but selective use of computation and digital measurement where they reduce a specific uncertainty.

This focus also creates a clearer distinction between TRD and depression more generally. A formulation developed for rapid rescue, maintenance therapy, anti-inflammatory adjunctive treatment, or cognitive symptoms will require different exposure profiles and outcome measures. The indication should therefore be specified before the material is optimized. Biomaterial design cannot compensate for an undefined therapeutic objective.

Several uncertainties remain. Direct nose-to-brain transport in humans is difficult to quantify, and animal nasal anatomy can exaggerate access to olfactory regions. Long-term effects of repeated nanoparticle exposure on olfactory tissue are incompletely characterized. Many candidate cargos have not established that improved regional delivery changes clinically important outcomes. In addition, some examples discussed in this Perspective are drawn from general depression, other central nervous system disorders, or broader intranasal delivery studies; these examples are used to support engineering principles and translational design logic rather than to imply direct therapeutic evidence for TRD. These limitations support a more disciplined program, not abandonment of the route.

The central conclusion is that intranasal biomaterial–device products for TRD should be developed as an exposure-engineering problem. The product is a coupled system, and every link from material structure to clinical outcome should be experimentally accountable. AI, medical imaging, CFD, 3D-printed models, and digital measures may accelerate selected steps, but the scientific value of this framework remains grounded in biomaterial transport, device–formulation performance, pharmacokinetics, safety, and therapeutic validation. By organizing development around reproducible therapeutic exposure rather than material sophistication alone, this approach may improve comparability across studies, support more rational product optimization, and provide a clearer translational pathway for intranasal therapies in treatment-resistant depression.

## Data Availability

The original contributions presented in the study are included in the article/supplementary material, further inquiries can be directed to the corresponding author.
